# Supplementary figures and images for: Comprehensive Characterization and Functional Analysis of the Lateral Organ Boundaries Domain Gene Family in Rice: Evolution, Expression, and Stress Response
Source: Int J Mol Sci. 2025 Apr 22;26(9):3948. doi: 10.3390/ijms26093948 (PMC12071882; doi:10.3390/ijms26093948)

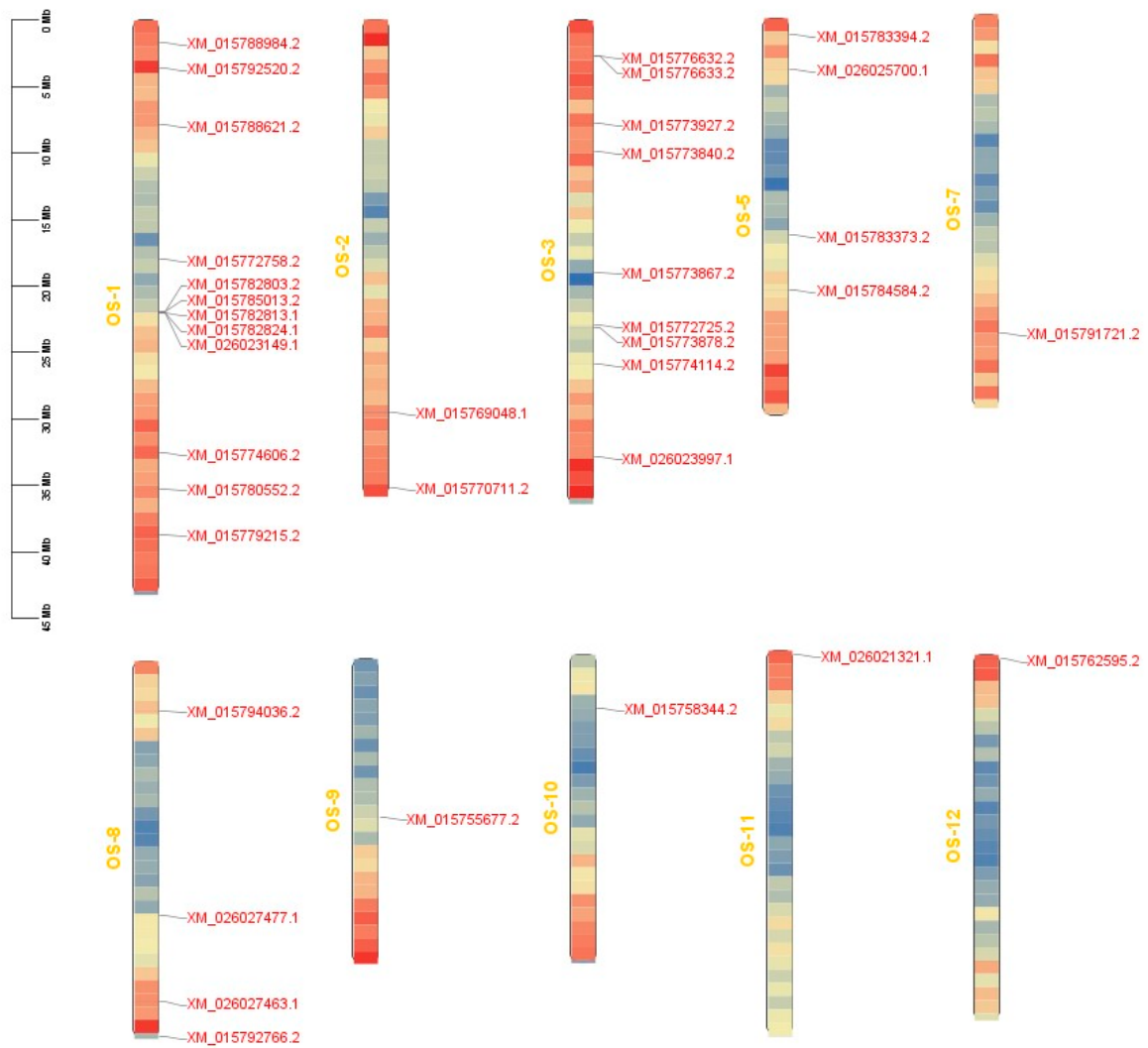

Figure S1:Chromosomal mapping of the LBD gene in rice.

Supplement: Supplementary file 1 [file ijms-26-03948-s001.zip › Figure S1.pdf]
